# Vascular plants of old cemeteries in the Lower Dnipro region (Southern Ukraine)

**DOI:** 10.3897/BDJ.11.e99004

**Published:** 2023-02-01

**Authors:** Nadiia Skobel, Ivan Moysiyenko, Barbara Sudnik-Wójcikowska, Iwona Dembicz, Maria Zachwatowicz, Maryna Zakharova, Oleksii Marushchak, Vikroria Dzerkal

**Affiliations:** 1 Kherson State University, Faculty of Biology, Geography and Ecology, Department of Botany, Kherson, Ukraine Kherson State University, Faculty of Biology, Geography and Ecology, Department of Botany Kherson Ukraine; 2 University of Warsaw, Faculty of Biology & Biological and Chemical Research Centre, Warsaw, Poland University of Warsaw, Faculty of Biology & Biological and Chemical Research Centre Warsaw Poland; 3 University of Warsaw, Faculty of Geography and Regional Studies, Warsaw, Poland University of Warsaw, Faculty of Geography and Regional Studies Warsaw Poland; 4 I. I. Schmalhauzen Institute of Zoology, NAS of Ukraine, Kyiv, Ukraine I. I. Schmalhauzen Institute of Zoology, NAS of Ukraine Kyiv Ukraine; 5 Nyzhnodniprovskyi National Nature Park, Kherson, Ukraine Nyzhnodniprovskyi National Nature Park Kherson Ukraine

**Keywords:** occurrence, refuge of steppe flora, floristic richness, grass steppe, Kherson Region, Ukraine.

## Abstract

**Background:**

The dataset contains the records of vascular plant species occurrences and distribution in old cemeteries (OC) of the Lower Dnipro region (Southern Ukraine). The analysed cemeteries were located in different types of landscapes (agricultural, rural and urban) and represent various ways of using their area (currently used, closed, abandoned). The floristic list includes 440 species of vascular plants (437 *in situ*, 3 *ex situ)*. The dataset demonstrates a sozological (Red-lists species) value of old cemeteries in Southern Ukraine. The cemeteries constitute refuges of native, rare and steppe flora and play a role of steppe habitat islands in a landscape almost completely transformed to arable land or urbanised.

**New information:**

This is the first dataset which contains information about flora of old cemeteries in Lower Dnipro region (Southern Ukraine). The dataset comprises 2118 occurrences of vascular plants (440 species) recorded in the years 2008-2021 in 13 old cemeteries of the Lower Dnipro region. The dataset includes information about 85 occurrences of rare species (23 species *in situ*, 3 *ex situ*) and 652 occurrences of 117 steppe species.

## Introduction

In recent centuries, anthropogenic activities led to significant losses of natural habitats, globally (Cremene et al. 2005, Vickery et al. 2009, Löki et al. 2019). Particularly significant changes occurred in the steppe zone of southern Ukraine, where the area of steppe vegetation decreased forty-fold in the beginning of the 19^th^ and 20^th^ century. The steppe covered ca. 40% of the total territory of the today’s Ukraine, while at present, the steppe remnants survived only on 1% of this area (Burkovskyi et al. 2013). In the steppe zone of Europe, small anthropogenic objects (Verschuuren et al. 2010, Valkó et al. 2018) like cultural monuments (Deák et al. 2016a, Deák et al. 2016b), ancient burial mounds (e.g. Moysiyenko and Sudnik-Wójcikowska (2006a), Moysiyenko and Sudnik-Wójcikowska (2006b), Sudnik-Wójcikowska and Moysiyenko (2006), Sudnik-Wójcikowska and Moysiyenko (2008a), Sudnik-Wójcikowska and Moysiyenko (2008b), Moysiyenko and Sudnik-Wójcikowska (2008), Moysiyenko and Sudnik-Wójcikowska (2009), Moysiyenko and Sudnik-Wójcikowska (2010), Sudnik-Wójcikowska and Moysiyenko (2010), Sudnik-Wójcikowska and Moysiyenko (2011a), Sudnik-Wójcikowska et al. (2011), Sudnik-Wójcikowska and Moysiyenko (2012), Moysiyenko et al. (2014)), old cemeteries (e.g. Verschuuren et al. (2010), Moysiyenko et al. (2017), Löki et al. (2019)) and ancient settlements (e.g. Dayneko et al. (2020), Dayneko (2020), Moysiyenko et al. (2020)) are often the last enclaves of steppe vegetation and constitute important sites for steppe biodiversity conservation.

The studies performed in different regions have recognised the natural values of cemeteries (e.g. Galera et al. (1993), Lisowska et al. (1994), Barrett and Barrett (2001), Vickery et al. (2009), Löki et al. (2019), Nowińska et al. (2019), Nowińska et al. (2020)); however, studies focused on the natural values of old cemeteries In Ukraine were lacking. There were a few publications devoted to the spontaneous and decorative flora of Ukrainian cemeteries, as well as some research that focused on urban areas (e.g. Burda (1991), Vasylieva-Nemertsalova (1996), Moysiyenko (1997), Moysiyenko (1999), Melnyk (2001), Arkushyna (2003a), Arkushyna (2003b), Hubar (2006), Kushnyr (2006), Hamulia and Zviahyntseva (2010), Zavialova (2010), Sukhanova (2012), Eremenko (2013), Gerasimyuk (2014), Besarabchuk and Volhin (2017), Maltseva (2019)). Given the extremely high level of anthropogenic transformation of the steppe flora of Ukraine, the role of the phytodiversity research on small objects, such as old cemeteries, is growing. Cemeteries can be treated as “islands” of natural vegetation located in close proximities of urban areas, often harbouring rare and endangered plant species. The key importance of cemeteries in nature conservation is unquestionable (Bhagwat 2009).

Using the term ‘old cemetery’, we refer to the European Landscape Convention (Council of Europe 2000), according to which, the old cemeteries are treated as cultural heritage sites and integral parts of the natural-cultural system. Importance of old cemeteries in the conservation of steppe flora of South Ukraine is highly dependent on the date when the cemetery was established. The establishment period in the mid-19^th^ to the early 20^th^ century or earlier is particularly relevant, since, at that time, the cemeteries were located within the natural steppe habitats, while later, the significant transformation of the steppe cover to arable land occurred.

One of the problems of the natural and cultural heritage conservation of old cemeteries is the fact that still none of the old cemeteries of Lower Dnipro region is included in the register of monuments of Ukraine. Thus, many of them could be neglected and destroyed (Ukraine Incognita 2022, Fig. 1).

Our field investigations, in the years of: 2007-2017 and 2020-2021, showed the capacity and value of old cemeteries for biodiversity conservation. The flora of the investigated old cemeteries included 440 taxa of vascular plants. The total number of occurrences was 2118. Amongst the recorded taxa, numerous were protected and Red-listed plant species (recorded in the Red Data Book of Ukraine, Didukh (2009)). Old cemeteries should be, therefore, the subjects of special protection as cultural and natural monuments. They constitute enclaves of steppe and rare species. It is suggested that active conservation measures in steppe burial sites should only take place if protection of the site cannot be ensured otherwise, for example, when a change in religious values results in a discontinuation of traditional habitat management methods (Löki et al. 2019). Conservation, history and spiritual activities are closely connected in cemeteries. However, establishment of protected areas in currently-used cemeteries is problematic at the moment, since the objectives of nature conservation sites and the traditions of burial care have not matched (and usually provide for the destruction of a vegetation cover). Therefore, for currently-used cemeteries, it is more challenging. Therefore, for current used, it is more advisable to apply a gentle informational campaign with explanation of ways of ecologisation of burial care. In contrast, abandoned cemeteries that have a high conservation value already could be declared natural monuments. According to Ukrainian law (in Ukrainian: ‘‘pamyatki prirody’’), they are protected by adequate environmental regulations including in its description ‘‘unique structures with exceptional natural, scientific, educational and aesthetic values that should remain intact’’.

### The goals that were set for the study

The floristic data, collected in the old cemeteries (OC) were compiled into a dataset. We used the dataset to achieve the following goals:

1. to characterise the floristic richness and value of old cemeteries in the Kherson Region, Southern Ukraine (on the example of selected cemeteries, representative of the region);
2. to indicate the most valuable species (steppe and rare species, legally protected or listed in the ’Red Data Book of Ukraine’ (Didukh 2009);
3. to show the role of old cemeteries in the preservation of the steppe flora.

## Project description

### Title

Northern Eurasia 2022

### Personnel

Nadiia Skobel, Ivan Moysiyenko

### Funding

The collecting of floristic data, field investigations and further data analysis were supported by the project:

‘How the East was won: Towards an environmental history of the Eurasian steppe’ N 2012-06112.

We are also grateful to the ‘Finnish Biodiversity Information Facility (FinBIF)’ for the call for authors in the project ‘Northern Eurasia 2022’ The data processing and publication were funded by the NCN scholarship programme for Ukrainian students and young researchers nr 2021/01/4/NZ9/00078 in collaboration with the University of Warsaw (N.S.).

## Sampling methods

### Study extent

See Moysiyenko et al. 2021a, Moysiyenko et al. 2021b, Moysiyenko et al. 2021c, Moysiyenko et al. 2021d, Skobel et al. 2022, Skobel and Moysiyenko 2022a, Skobel and Moysiyenko 2022b, Skobel et al. 2022a, Skobel et al. 2022b. According to the administrative and territorial division, the examined cemeteries are located in the Beryslav, Henichesk, Skadovsk and Kherson Districts (former Belozerka, Beryslav, Velykooleksandrivka, Hola Prystan and Nyzhni Sirogozy Districts) and Kherson — the capital of the Kherson Region. The study of the flora of 13 old cemeteries was conducted using literature and field data, collected during the growing seasons of 2008–2021. The study of the flora of 10 old cemeteries of the Kherson Region (its rural and agricultural landscape) was conducted in the growing seasons of 2008-2017, field searching during two-thee years and three old cemeteries of the city of Kherson (urban areas) in 2020-2021 field searching during two years.

The area of the old cemeteries of the Kherson Region varies from 0.43 ha to 10.45 ha. The total area of all old cemeteries is 51.28 ha (Fig. 2,Fig. 3, Tables 1, 2, 3).

### Sampling description

We were guided by the following criteria for selecting old cemeteries for research:

a) the establishment of the old cemetery in the areas covered by steppe sites (cemeteries established by the beginning of the 20^th^ century and earlier). There is no register of old cemeteries available in Ukraine. By this reason, we used literature sources (Kasyanenko 1972) and historical maps (Maps of Schubert 1865) to search for old cemeteries and for estimations of the year of their foundation.

b) the presence of preserved steppe sites of more than 10 m^2^ (within the cemetery area and around it).

c) the presence of rare plant species and plant communities.

All analysed cemeteries are located in different types of landscapes: agricultural, rural and urban (Fig. 3).

Rural cemeteries are located in villages, within rural landscapes. Rural landscape describes the diverse portion of the nation's land area not densely populated or intensively developed and not set aside for preservation in a natural state. Rural landscapes have differences in comparison with urban; as rule in rural, they have less anthropogenic territories and activities, while agricultural cemeteries are located in agricultural landscape between ploughed areas of fields. Agricultural landscape features (or henceforward simply landscape features) are small fragments of non-productive natural or semi-natural vegetation in an agricultural landscape which provide ecosystem services and support for biodiversity (European Commission, Joint Research Centre 2022).

a) currently used OC – intensive land use (burials, intensive planting, intensive care of graves, littering, possible grazing, absence of mowing);

b) closed OC – medium land use intensity (burials not performed, medium planting, medium care of graves, possible littering, possible grazing, possible mowing);

c) abandoned OC – relatively medium/low land use intensity (burials not performed, low planting, possible care of graves, low littering, possible grazing, possible mowing).

As rural and agricultural cemeteries were often anonymous, we called them according to the name of the nearest village or historical place (in case the nearest village was absent).

Each old cemetery was examined at least three times during the growing season (spring, summer and autumn) using the route-field method. The floristic lists were collected. We determined the abundance of individual species according to a simple 3-point scale, where 1 means - single occurrence, 2 - several localities, 3 - quite common species within the site. The identification of vascular plant species was held in the field. Specimens that could not be identified in the field were collected to the Kherson State University Laboratory of Plant Ecology and Environmental Protection. The floristic lists were the subject to further analysis (Moysiyenko et al. 2021a, Moysiyenko et al. 2021b, Moysiyenko et al. 2021c, Moysiyenko et al. 2021d, Skobel et al. 2022, Skobel and Moysiyenko 2022a, Skobel and Moysiyenko 2022b, Skobel et al. 2022a, Skobel et al. 2022b).

The collective list of old cemeteries flora includes 437 species *in situ* and three rare species *ex situ* (i.e. native plants which escaped cultivation after they were intentionally brought to the cemetery). The species cultivated on graves, which have not gone wild, were not included in the species lists.

### Quality control

The collected materials were verified in the Laboratory of Plant Ecology and Environmental Protection (Department of Botany, KSU) and Herbarium of Kherson State University (KHER, Kherson State University 2022). Species identification extracted from peer-reviewed scientific publications were taken as is, but checked for name misspelling against GBIF Species Matching tool. Coordinates of records were checked using Google Earth service (Google Earth 2021) and QGIS.

### Step description

The following steps were taken:

1. The study of vascular plant flora in old cemeteries of the Lower Dnipro region was carried out in the field, in the growing seasons of 2007-2018 and 2020-2021.
2. We collected the lists of species and determined the abundance of individual species in each cemetery according to a simple 3-point scale.
3. To make the lists of the flora comparable, we strived to visit each of the old cemeteries at different times of the growing season (spring, summer, autumn). Thus, the floristic lists were successively supplemented.
4. We collected herbarium documentation (Kherson State University 2022) and photographic documentation.
5. The obtained census of the old cemeteries flora includes 440 species and 2118 occurrences compiled in a CSV file.
6. Data were post-processed using Darwin Core terms (Wieczorek et al. 2012).
7. Data cleaning was performed using OpenRefine (OpenRefine 2022).

## Geographic coverage

### Description

The floristic research was carried out on 13 old cemeteries in the Lower Dnipro Province, Kherson Region. According to the administrative and territorial division, the examined cemeteries are located in the Beryslav, Henichesk, Skadovsk and Kherson Districts and in the city of Kherson.

The Lower Dnipro region is represented by classes of steppe vegetation (Sudnik-Wójcikowska and Moysiyenko 2011a, Sudnik-Wójcikowska and Moysiyenko 2011b, Sudnik-Wójcikowska et al. 2011). The climate is continental, with a mean annual temperature of 9–10°C. The characteristic features of the natural conditions of the Lower Dnipro region are determined by its geographical location within the true steppe zone of the Eastern European plain. The climate of the Lower Dnipro is temperate-continental with mild snowless winters and hot dry summers. The total annual precipitation is below 350 mm. Loess is the most common geological surface formation in the region, reaching a thickness of several tens of metres. Under the loess lies Neogene deposits (limestone, sands, sandstones, marls and clays) in the whole territory along the Lower Dnipro. A dominating undulating topography sets it significantly apart from surrounding steppe plains. The soil types represented within the study area are: low-humus chernozem, dark chestnut, sod and clay sand and meadow-swamp soils (Marynych and Shyshchenko 2005).

The land-surface is almost flat. The Dnipro floodplain is densely cut by numerous straits and branches. Regarding the geobotanical zoning, the Lower Dnipro region is located in three districts of the Black Sea and Azov steppe sub-province of the Pontic steppe province of the steppe zone: a) Bug-Ingul District of grasses, grass meadows and vegetation of limestone outcrops; b) Lower Dnipro region of sand steppes, sands and reed beds; c) Dnipro-Azov District of grasses, wormwood-grass steppes and depression (pid – in Ukrainian) meadows (Andrienko et al. 1977, Dayneko 2020, Moysiyenko et al. 2021a).

### Coordinates

46.134 and 47.48 Latitude; 31.904 and 35.057 Longitude.

## Taxonomic coverage

### Description

The Scientic Names of species are given in Latin according to Vascular Plants of Ukraine, a nomenclatural checklist (Mosyakin and Fedoronchuk 1999). The total list of flora *in situ* include 437 species and, additionally, the dataset includes information about three rare species *ex situ* (planting and wilding of rare species which is one of typical traits of flora of old cemeteries in the Lower Dnipro region and for by this reason, we did not include cultivated species because, as rule, these species donot indicate a high level of biodiversity). In total, all species were identified to 440 species. All occurrences classified to one phylum Tracheophyta, to three classes (Gnetopsida, Liliopsida, Magnoliopsida), 27 orders Apiales, Asparagales, Asterales, Boraginales, Brassicales, Caryophyllales, Cucurbitales, Dipsacales, Ephedrales, Ericales, Fabales, Fagales, Gentianales, Geraniales, Lamiales, Liliales, Malpighiales, Malvales, Poales, Ranunculales, Rosales, Santalales, Sapindales, Saxifragales, Solanales, Vitales, Zygophyllales) and 61 families (Adoxaceae, Amaranthaceae, Amaryllidaceae, Apiaceae, Apocynaceae, Asparagaceae, Asphodelaceae, Asteracae, Berberidaceae, Betulaecea, Bignoniaceae, Boraginaceae, Brassicaceae, Campanulaceae, Cannabaceae, Caprifoliaceae, Caryophyllaceae, Convolvulaceae, Crassulaceae, Cucurbitaceae, Cyperaceae, Elaeagnaceae, Ephedraceae, Euphorbiaceae, Fabaceae, Fagaceae, Geraniaceae, Grossulariaceae, Heliotropiaceae, Hypericaceae, Iridaceae, Juglandaceae, Lamiaceae, Liliaceae, Linaceae, Malvaceae, Moraceae, Oleaceae, Paeoniaceae, Papaveraceae, Plantaginaceae, Plumbaginaceae, Poaceae, Polygonaceae, Portulacaceae, Primulaceae, Ranunculaceae, Resedaceae, Rhamnaceae, Rosaceae, Rubiaceae, Salicaceae, Sapindaceae, Scrophulariaceae, Simaroubaceae, Solanaceae, Thesiaceae, Ulmaceae, Violaceae, Vitaceae, Zygophyllaceae).

### Taxa included

**Table:** 

Rank	Scientific Name	
kingdom	Plantae	

## Temporal coverage

**Data range:** 2008-1-01 – 2017-12-31; 2020-1-01 – 2021-12-31.

## Usage licence

### Usage licence

Open Data Commons Attribution License

### IP rights notes

This work is licensed under a Creative Commons Attribution (CC-BY) 4.0 Licence.

## Data resources

### Data package title

Vascular plants of old cemeteries of Lower Dnipro region (Southern Ukraine)

### Resource link

https://doi.org/10.15468/h82vw6

### Alternative identifiers

https://ukraine.ipt.gbif.no/resource?r=vp_cemeteries

### Number of data sets

1

### Data set 1.

#### Data set name

Vascular plants of old cemeteries of Lower Dnipro region (Southern Ukraine)

#### Data format

Darwin Core

#### Download URL

https://www.gbif.org/uk/dataset/4f5a8595-6bda-4a3b-9d07-c0cdc38ffdef

#### Description

The dataset includes a table with 29 fields in Darwin Core terms and 2118 records in it (Skobel et al. 2023).

**Data set 1.** 

Column label	Column description
occurrenceID	An identifier of a particular occurrence, unique within this dataset. We used the species occurrence numbers.
scientificName	The original names according to ‘Vascular Plants of Ukraine, a nomenclatural checklist’ (Mosyakin, Fedoronchuk 1999), corrected for spelling mistakes using GBIF Species Matching tool.
eventDate	The date-time or interval during which an Event occurred.
basisOfRecord	The method in which data were acquired (HumanObservation).
geodeticDatum	The geodetic datum upon which the geographic coordinates are given. In our case, it is always WGS84.
georeferencedBy	Theperson who determined the georeference.
georeferenceProtocol	A description of the method used to determine coordinates (Manual with Google Earth).
recordedBy	The persons who is responsible for recording the original Occurrence.
identifiedBy	The persons who is responsible for recording the Taxon to the subject.
coordinateUncertaintyInMetres	The distance (in metres) from the given decimalLatitude and decimalLongitude describing the smallest circle containing the whole of the Location (from 43 m to 278 m).
geoReferenceRemarks	Notes about the spatial description determination, explaining assumptions made in addition or opposition to the those formalised in the method referred to in georeferenceProtocol (describing the smallest circle containing the whole of the Location (from 43 m to 278 m).
decimalLatitude	The geographic latitude in decimal degrees.
decimalLongitude	The geographic longitude in decimal degrees.
organismQuantity	A number or enumeration value for the quantity of organisms. Estimated according to a 3-point scale: 1 – sporadic, 2 – fairly frequent, 3 – common.
organismQuantityType	The type of quantification system used for the quantity of organisms. We used a 3-point scale.
samplingProtocol	The names of the method used during an Event (Species Shoot Presence).
countryCode	The standard code for the country in which the Location occurs. In our case, it is always UA.
country	The name of the country unit in which the Location occurs. In our case, it is always Ukraine.
stateProvince	The name of the administrative region of Ukraine in which the Location occurs. In our case, it is always Kherson.
county	The full, unabbreviated name of the next smaller administrative region than stateProvince (districts).
locality	The specific description of the place. The nearest village and name of cemetery or official name of cemetery.
taxonRank	The taxonomic rank of the most specific name in the scientificName.
kingdom	The full scientific name of the kingdom in which the taxon is classified. In our case, it is always Plantae.
phylum	The full scientific name of the phylum or division in which the taxon is classified. In our case, it is always Tracheophyta.
class	The full scientific name of the class in which the taxon is classified. In our case, it is Magnoliopsida, Liliopsida, Gnetopsida.
order	The full scientific name of the order in which the taxon is classified. In our case, it is Asterales, Lamiales, Caryophyllales etc. (fig. 5; Taxonomic distribution of occurrences).
family	The full scientific name of the family in which the taxon is classified (fig. 5 Taxonomic distribution of occurrences).
recordedByID	A list of the globally unique identifiers for the people responsible for recording the original Occurrence.
identifiedByID	A list (concatenated and separated) of the globally unique identifiers for the people responsible for assigning the Taxon to the subject.

## Additional information

### Floristic richness and taxonomic value of old cemeteries in the Lower Dnipro region

We identified 437 (+3 *ex situ*) taxa of vascular plants in 13 old cemeteries, which make up 8.5% of the total flora of Ukraine (Mosyakin and Fedoronchuk 1999) and 21.5% of the flora of the Northern Black Sea region (Moysiyenko 2013).

The total species richness ranged from 104 to 217 (162 species per one cemetery in average). Old cemeteries in different types of landscape show different values of floristic richness.

We noticed differences in floristic richness depending on cemetery size (Fig. 4, Table 2, Moysiyenko et al. (2021a), Moysiyenko et al. (2021b), Moysiyenko et al. (2021c), Moysiyenko et al. (2021d), Skobel et al. (2022), Skobel and Moysiyenko (2022a), Skobel and Moysiyenko (2022b), Skobel et al. (2022a), Skobel et al. (2022b)). Cemetery size can affect the floristic richness. Larger cemeteries should have a higher degree of species richness (Nowińska et al. 2019, Nowińska et al. 2020). Articles of Nowinska (Nowińska et al. 2019, Nowińska et al. 2020) show more conserved values of the older cemeteries than the earlier age of establishment in Europe. Old cemeteries in Lower Dnipro region is related to their location in landscape, than the age of old cemeteries. In southern Ukraine, for a long time, from the mid-19^th^ to the early 20^th^ century, only grazing was the predominant type of land use and all old cemeteries were founded before the massive ploughing of the steppes; for this reason, the age of the old cemeteries does not significantly impact their flora.

At the same time, although Kherson city's old cemeteries are covering larger areas (6.5 ha on average) compared to cemeteries located in other types of landscape (2.8 ha on average), they are poorer regarding species diversity and more homogenous (floristically similar to each other – they share 125 species, 55%). This is due to anthropogenic impacts occurring in the City, the similar exotic species occurrences and the relatively small distances between cemeteries in the City (the distance between cemeteries located in Kherson City is ca. 2.5 km, while, for rural landscapes, this value ranges from 1.5 km to 140.5 km).

The way of using cemeteries’ areas affects their floristic diversity. Currently, used cemeteries are richer in species and more diverse than abandoned ones, because human activities (burials, ornamental species introductions etc.) usually contribute to the short-term emergence of random species (Nowińska et al. 2020, Nowińska et al. 2019). The examined cemeteries seem to confirm this pattern (Table 2), but a greater number of sampled cemeteries would be needed in order to confirm it as a rule.

The location of the cemetery in a more or less transformed landscape significantly influences its flora. Old cemeteries in the city of Kherson (urban landscape) are characterised by high shares of alien species, compared to old cemeteries in rural and agricultural areas. It can be explained by the specific, anthropogenic impacts. Human activities in the cities result in a high input of propagules and, in consequence, also in a significant number of alien species (Kornaś 1981, Pyšek 1998, Protopopova 1991, Rejmánek 2000, Protopopova and Shevera 2005, Kowarik 2008, Lososová et al. 2012a, Lososová et al. 2012b, Török et al. 2016).

The flora of investigated old cemeteries has a significant share of plants escaping from cultivation (Moysiyenko et al. 2021a, Moysiyenko et al. 2021b, Moysiyenko et al. 2021c, Moysiyenko et al. 2021d, Skobel et al. 2022, Skobel and Moysiyenko 2022a, Skobel and Moysiyenko 2022b, Skobel et al. 2022a, Skobel et al. 2022b). This is due to the intensive planting of ornamental plants on graves (the ornamental plants are represented by both non-native and native species).

The absence of ornamental plants can impact on the more natural vegetation in cemeteries. Given that ornamental plants are often cultivated on graves, their shares in the cemetery's flora is sometimes high and, thus, the risk of plant invasions is also high for this type of habitat. In such conditions, many sensitive native species, especially steppe species, are unable to compete with more successful non-native plants (Löki et al. 2019).

From the perspective of preservation of a natural vegetation cover, widespread cultivation of plants in cemeteries has two consequences. Wild non-native plants show a negative effect by competing with local plants. In particular, large areas of neglected cemeteries are occupied by shrubs (e.g. *Syringavulgaris*, *Ailanthusaltissima*, *Lyciumbarbatum*). Some native woody plants (trees, such as: *Fraxinusexcelsior*, *Quercusrobur* and shrubs, such as *Ligustrumvulgare*) are native, but not characteristic of the steppe vegetation and are also cultivated in cemeteries and escape from cultivation. They may have a negative impact on steppe vegetation in cemeteries and in their vicinity (Sudnik-Wójcikowska and Moysiyenko 2012). On the other hand, ornamental native, especially steppe species, intentionally planted in cemeteries, have a chance to survive and spread locally, even outside the cemetery.

Most of the species (42%) appeared only in 1-2 cemeteries (Fig. 5). This can be explained by the behaviour of visitors who deliberately bring and plant plants or accidentally drag the diasporas. The number of the species belonging to the I^st^ class of frequency is 184 (they are usually ephemerophytes, ergasiophytes and ergaziophygophytes).

The majority of species (99.8%) belong to Magnoliophyta division (Fig. 6). The division Pinophyta (0.2%) is represented by one family – Ephedraceae and one species – *Ephedradistachya* L. The presence of Lycopodiophyta, Polypodiophyta and Equisetophyta in the flora of cemeteries was not confirmed, which is explained by unfavourable environmental conditions of the steppe zone (in particular, the insufficient level of moisture).

The most represented families of class Magnoliophyta in the old cemeteries flora are: Asteraceae, Poaceae and Fabaceae. These families are also well represented in the flora of Ukraine and within the flora of kurgans (Moysiyenko and Sudnik-Wójcikowska 2006a, Moysiyenko and Sudnik-Wójcikowska 2006b, Sudnik-Wójcikowska and Moysiyenko 2006, Sudnik-Wójcikowska and Moysiyenko 2008a, Sudnik-Wójcikowska and Moysiyenko 2008b, Moysiyenko and Sudnik-Wójcikowska 2009, Sudnik-Wójcikowska and Moysiyenko 2010, Sudnik-Wójcikowska and Moysiyenko 2011a, Sudnik-Wójcikowska and Moysiyenko 2011b, Sudnik-Wójcikowska and Moysiyenko 2012, Moysiyenko et al. 2014, Moysiyenko et al. 2023), ancient settlements (Dayneko 2020, Dayneko et al. 2020, Dayneko et al. 2023) and ancient parks (Khodosovtsev et al. 2019). On the other hand, the Rosaceae family (22 species) in old cemeteries is highly represented, mainly by phanerophytes – 16 species (Moysiyenko et al. 2021a, Moysiyenko et al. 2021b, Moysiyenko et al. 2021c, Moysiyenko et al. 2021d), which are not typical for the steppe flora (Krasnova A.M 1973, Krytska 1985, Kucherevskyi 2004, Bondarenko 2015, Shapoval 2012). Their occurrence can be related to local traditions, burial activities and grave care. The genera with the highest number of species identified in the old cemeteries were: *Veronica* (10), *Euphorbia* (8), *Prunus* (8), *Artemisia* (7), *Astragalus* (7), *Allium* (6), *Atriplex* (6), *Bromus* (6), *Galium* (6), *Medicago* (6), *Silene* (6), *Achillea* (5), *Carex* (5), *Chenopodium* (5), *Limonium* (5), *Potentilla* (5), *Sysimbrium* (5), *Vicia* (5), *Viola* (5), *Amaranthus* (4), *Centaurea* (4), *Iris* (4) and *Trifolium* (4).

### The richness of the native flora in old cemeteries of the Lower Dnipro region

The significant shares of native plants indicate a high level of preservation of vegetation in old cemeteries (Fig. 7). Most of the species were natives (272 species – 62.4% of the flora). More than half of the native species are non-synanthropic plants – 142 species. The V class of frequency of native species in old cemeteries include: *Arenariauralensis* Pall. ex Spreng., *Artemisiaaustriaca* Jacq., *Consolidapaniculata* (Host) Schur., *Convolvulusarvensis* L., *Coronillavaria* L., *Falcariavulgaris* Bernh., *Festucavalesiaca* Guadin, *Galiumaparine* L., *Holosteumumbellatum* L., *Medicagofalcata* L., *Poaangustifolia* L., *Poabulbosa* L., *Polygonumaviculare* L. s.str., *Potentillaargentea* L., *Pterothecasancta* (L.) C.Koch, *Seneciovernalis* Waldst. et Kit., *Seselitortuosum* L., *Taraxacumerythrospermum* Andrz., *Tragopogonmajor* Jacq., *Valerianellacarinata* Loisel. and *Violakitaibeliana* Roem. et Schult. The shares of native species in old cemeteries varied from 50.4% (Kherson Memorial сemetery) to 76.9 % (kurgan near khutir Balakshova).

Individual old cemeteries in urban landscape (nos. 1, 2 and 3) are also closed cemeteries. They all show a much smaller share of native species than old cemeteries in agricultural and rural landscapes. The three cemeteries richest in native species are located amongst fields, two of them being abandoned (nos. 4 and 5) and one (no. 11) is still in use (Fig. 7, Table 2). The way of using cemetery area affects the specificity of its flora. This applies in particular to abandoned cemeteries (Nowińska et al. 2019, Nowińska et al. 2020), which are characterised by lower species similarity and higher proportion of native plants than cemeteries that are still in use. The latter ones are richer in species and more diverse than abandoned ones due to human activities (such as burials, introduction of ornamental species etc.) which usually contribute to the short-term emergence of random species (Nowińska et al. 2019, Nowińska et al. 2020). With regard to the Lower Dnipro region, the observed patterns seem to be similar, but due to high diversity of sampled sites, a greater number of cemeteries would be needed in order to confirm it as the rule.

### Floristic richness of the steppe species in old cemeteries in Lower Dnipro region

According to the geobotanical division of the Eurasian Steppe Zone, the Lower Dnipro region is located in the Black Sea and Azov sub-province of the Pontic steppe province. The steppe physiognomy in the region is determined by tussock grasses of the genera *Stipa*, *Festuca*, *Koeleria* and *Agropyron* (Andrienko et al. 1977, Bohn et al. 2000). Old cemeteries harbour a number of steppe plant species. Old cemeteries (just as kurgans) are more stable habitats than, for example, roadside verges and field margins, thus they play a very important conservational role (Molnár et al. 2017). Older habitats have the potential to hold more grassland (i.e. steppe) species compared to road verges in Central European forest steppe areas (Deák et al. 2020).

The old cemeteries of the Lower Dnipro region preserve steppe vegetation. Steppe vegetation can be preserved only at cemeteries historically located on unploughed parts of the steppe. If the newly-created cemeteries are located in a heavily transformed habitat, the chances of survival for steppe species are much lower.

Lower Dnipro region represented by classes of steppe vegetation (Sudnik-Wójcikowska and Moysiyenko 2011a, Sudnik-Wójcikowska and Moysiyenko 2011b, Sudnik-Wójcikowska et al. 2011, Dayneko et al. 2020). According to the classification of Syntaxonomy of Ukrainian vegetation (Solomakha 2008) in the investigated old cemeteries of the Lower Dnipro region, the classes of steppe vegetation (*Festuco-Brometea* Br.-Bl. et Tx.ex Soó: steppes) and classes that are close to steppe vegetation (*Helianthemo-Thymetea* Romaschenko, Didukh et V. Sl.: steppe calciphilous communities; *Festuceteavaginatae* Soó ex Vičherek; steppe psammophytic communities) are present. According to the classification of Vegetation of Europe (Mucina et al. 2016) in old cemeteries of Lower Dnipro region, *Festuco-Brometea* Br.-Bl. et Tx.ex Soó is present.

*Helianthemo-Thymetea* Romaschenko, Didukh et V. Sl. was used for species that have an optimum occurrence on limestone outcrops, which are present in the Kherson Region and are represented by the diagnostic species *Achillealeptophylla* M.Bieb., *Euphorbiaglareosa* M.Bieb. and *Thymusdimorphus* Klokov & Des.-Shost. *Festuceteavaginatae* Soó ex Vičherek was used for species that have optimal occurrence on psamopthytic steppes, which are present in the Kherson Region and are represented by the diagnostic species Astragalus varius S.G.Gmel., *Erophilaverna* (L.) DC., *Euphorbiaseguierana* Neck, *Gypsophilapaniculata* L. and *Helichrysumarenarium* (L.) Moench.

Representatives of dianostic species of these classes have the status of specialists – typical ‘steppe species’, all other species being general specialist or ’non-steppe species’. At old cemeteries of the Lower Dnipro region, we identified 117 steppe specialіsits and 320 generalists.

As in the case of native species, the poorest in steppe species turned out to be closed urban cemeteries (nos. 1, 2 and 3). On the other hand, the largest share of steppe species was found for three cemeteries, two of which were mid-field (nos. 5 and 11) and one was rural (no. 13). Of these, one was abandoned and the rest are still in use (Fig. 8, Table 2).

The steppe species richness of the investigated old cemeteries is related to their location in the landscape. The old cemeteries in agricultural and rural landscapes are richer in ‘steppe species’ compared to the urban landscape type (Fig. 8). This is associated with lower synanthropisation of the flora and lower anthropogenic impacts in the rural and agricultural landscape types (which allowed for the persistence of some steppe sites). The steppe species are, in general, not dominant in old cemeteries and they occupy up to one third of the cemetery flora. The synanthropic species prevailing in the old cemetery flora are not typical for the steppe and are much more competitive than sensitive steppe species (Sudnik-Wójcikowska and Moysiyenko 2012).

In the V class of frequency (present at 10-13 old cemeteries) and also in typical “steppe species” *Agropyronpectinatum* (M.Bieb.) P.Beauv., *Carduusuncinatus* M.Bieb., *Carexstenophylla* Wahlenb., *Festucavalesiaca* Guadin, *Koeleriacristata* (L.) Pers., *Potentillarecta* L., *Ranunculusoxyspermus* M.Bieb., *Stipacapillata* L., *Taraxacumerythrospermum* Andrz. and *Vincaherbacea* Waldst. et Kit. occurred.

The Lower Dnipro region has a traditional custom of planting the ornamental steppe plants on the graves. Such plants often ‘escape’ and start growing spontaneously (e.g. *Stipacapillata* L., *Ficariacalthifolia* Rchb., *Irispumila* L, *Ornithogalumkochii* Parl., *Vincaherbacea* Waldst. & Kit. and *Violaodorata* L.). Some other beautiful flowering local plants are not planted near the graves, but appear there spontaneously and are not destroyed during the clearing of the graves from ’weeds’ (e.g. *Asparagusofficinalis* L., *Potentillarecta* L. and *Salvianemorosa* L; (Moysiyenko et al. 2021a, Moysiyenko et al. 2021b, Moysiyenko et al. 2021c, Moysiyenko et al. 2021d, Skobel et al. 2022, Skobel and Moysiyenko 2022a, Skobel and Moysiyenko 2022b, Skobel et al. 2022a, Skobel et al. 2022b). As a result, these species have a good chance of being preserved in the conditions of loss of their natural habitats.

### Rare species in the flora of old cemeteries

Under the conditions of human pressure on the natural vegetation, it is particularly important to ensure the protection of rare species and their diversity. The old cemeteries of the Lower Dnipro region preserve rare steppe species that have survived *in situ* for centuries. It was possible because the burial places in Ukraine are held in esteem and the maintenance activities are traditional, limited to the care of graves. One of the indicators of the conservation value of old cemeteries is the share of rare species (sozophytes) in their flora (Moysiyenko et al. 2017, Moysiyenko et al. 2022). The rare species were present in all 13 old cemeteries.

**Figure 1.**
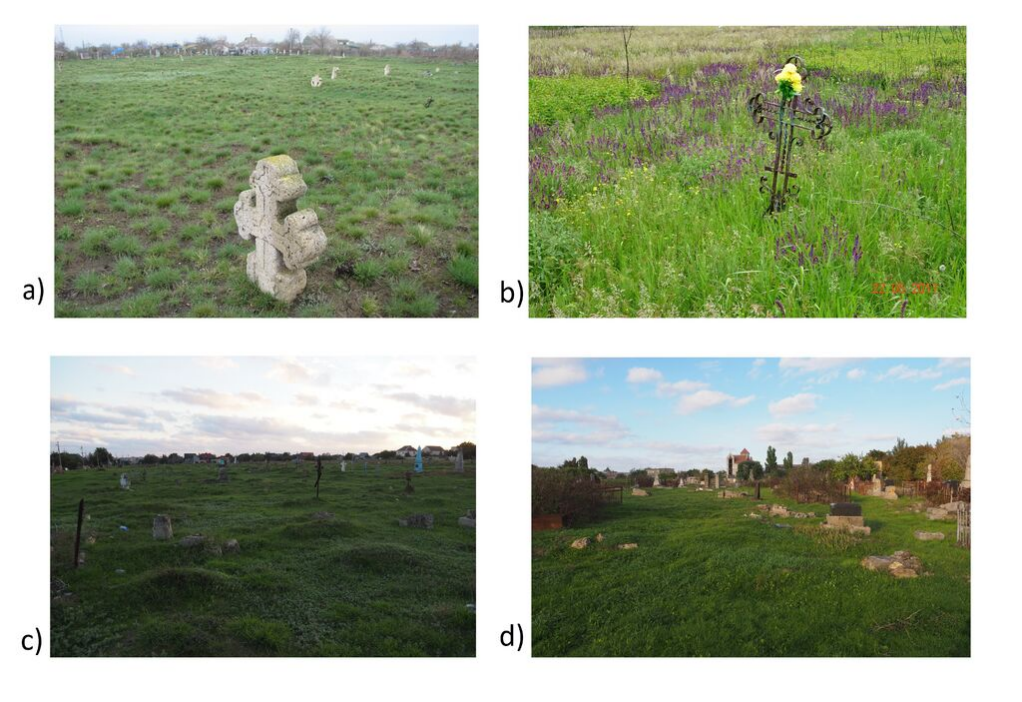
Grasslands in old cemeteries of the Lower Dnipro region: **a** Tiahynka; **b** Tryfonivka; **c** Kherson Zabalka cemetery, **d** Kherson Old Jewish cemetery.

**Figure 2.**
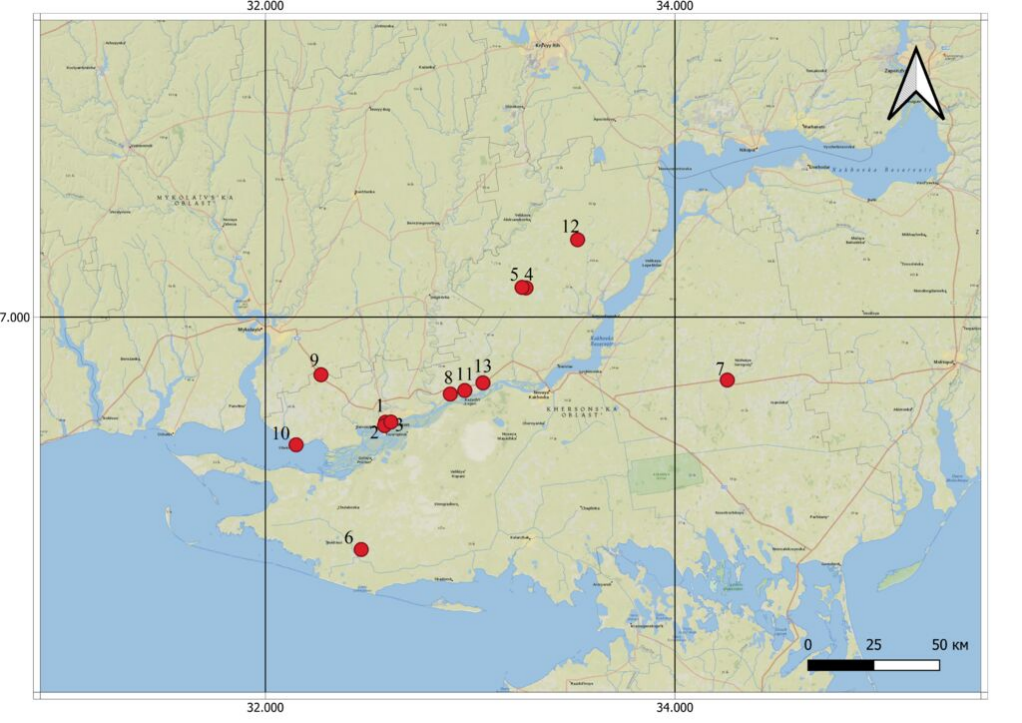
Location of old cemeteries. Abbreviations: 1 – Kherson Old Jewish cemetery; 2 - Kherson Zabalka cemetery; 3 – Kherson Memorial cemetery; 4 - Ekonomiia Ivanivka; 5 - Kurgan near khutir Balakshova; 6 – Dolmativka; 7 - Nyzhni Torhai; 8 – Poniativka; 9 - Posad-Pokrovske; 10 – Stanislav; 11 – Tokarivka; 12 – Tryfonivka; 13 – Tiahynka.*^1^

**Figure 3.**
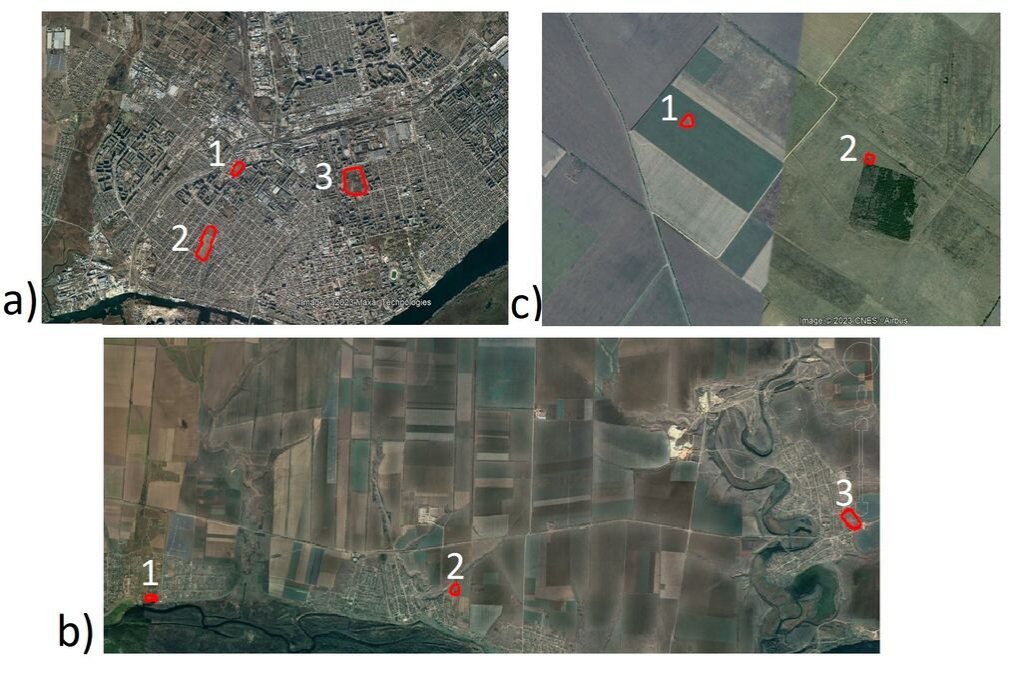
Satellite images of different types of landscape at the old cemeteries. Abbreviations: **a** – urban (1 – Kherson Old Jewish cemetery; 2 – Kherson Zabalka cemetery, 3 - Kherson Memorial cemetery); **b** – rural landscape (1 – Poniativka; 2 - Tokarivka; 3 - Tiahynka), **c** - agricultural landscape (1 - Kurgan near khutir Balakshova; 2 - Ekonomiia Ivanivka).

**Figure 4.**
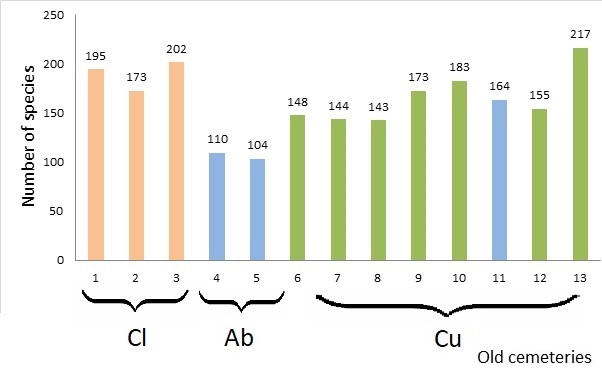
The total number of species in the flora of each of the 13 cemeteries (the number of species is indicated at the top of the bar). Abbreviations*^1^, ^3^, ^4^.

**Figure 5.**
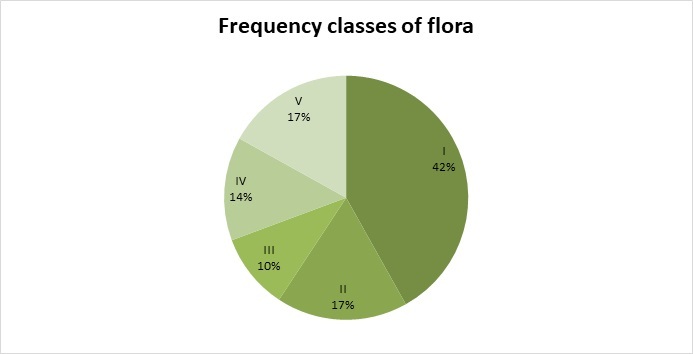
The total flora of old cemeteries by frequency classes (percentage of species in each category is indicated). Frequency classes: I – rare (1–2 cemeteries), II – relatively rare (3–4 cemeteries), III – not rare (5–6 cemeteries), IV – relatively frequent (7–9 cemeteries), V – common (10–13 cemeteries).

**Figure 6.**
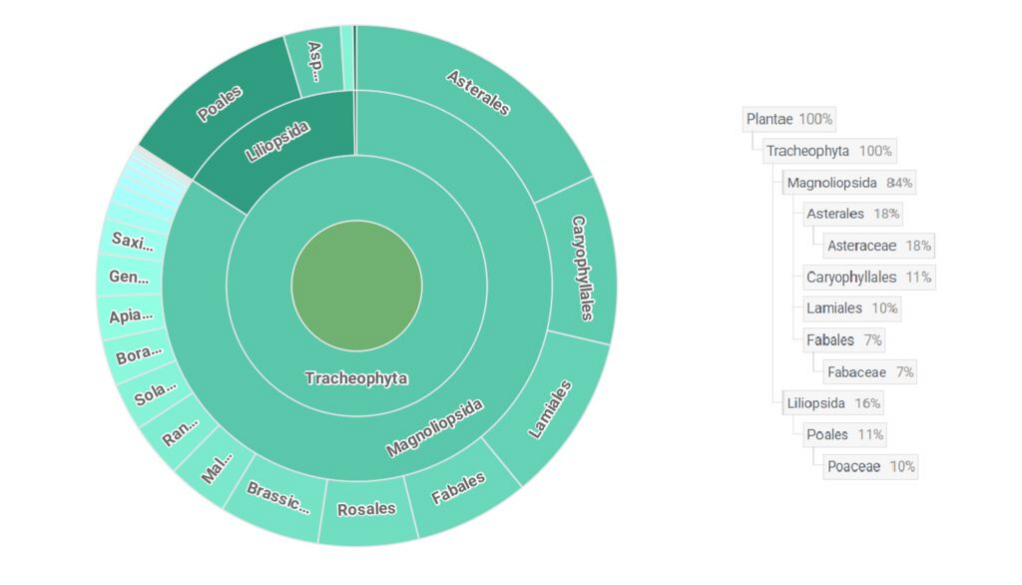
The taxonomic distribution of occurrences.

**Figure 7.**
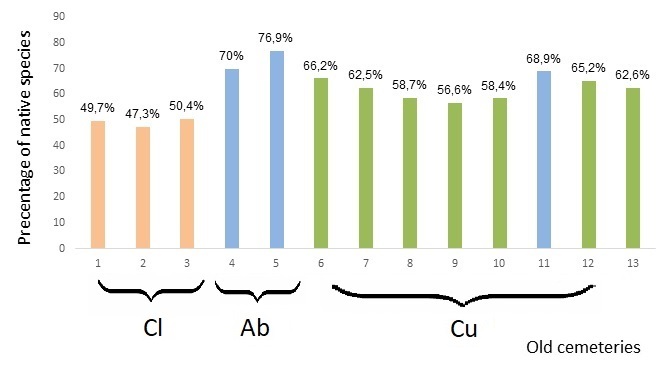
The shares of native species in the total flora of investigated old cemeteries. Abbreviations*^1^, ^3^, ^4^

**Figure 8.**
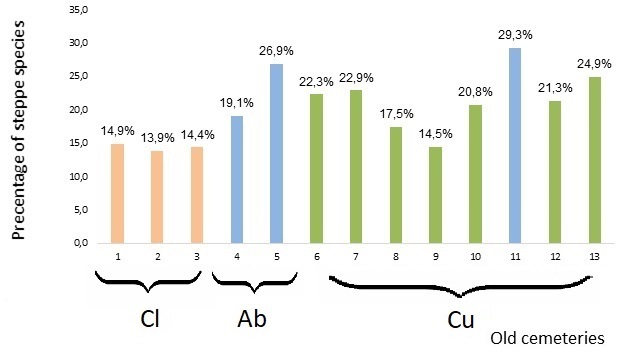
The shares of the ‘steppe species’ in the total flora of old cemeteries. Abbreviations*^1^, ^3^, ^4^.

The dataset includes information on 26 rare species in old cemeteries (5.2%). The cemeteries are usually small in size and isolated by agricultural, rural or urban areas around them (Fig. 1).

**Figure 9.**
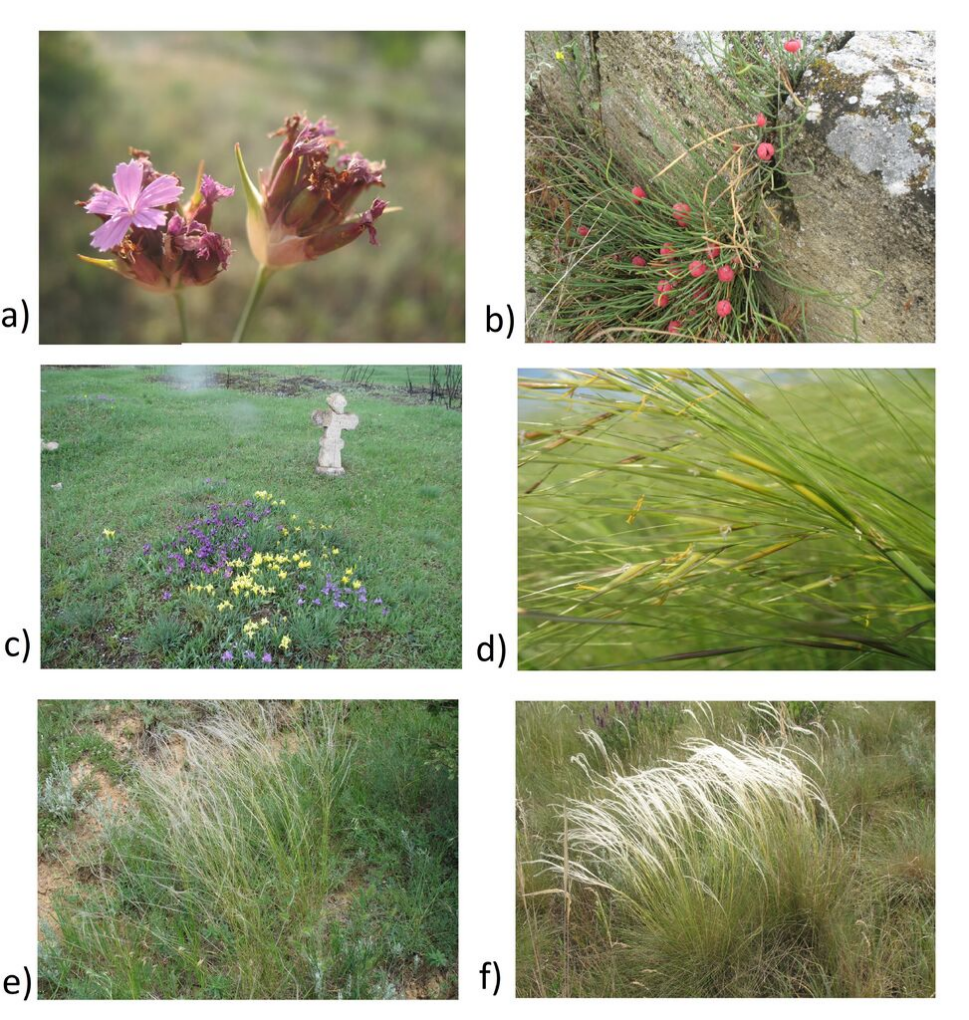
Rare steppe species on some old cemeteries of the Lower Dnipro**. a** *Dianthusandrzejowskianus*; **b** *Ephedradistachya*; **c** *Irishalophyla*; **d** *Stipacapillata*; **e** *Stipalessingiana*; **f** *Stipaucrainica* (photo Ivan Moysiyenko)

Six vascular plant species are included in the Red Data Book of Ukraine (Didukh 2009), i.e. *Astragalushenningii* (Steven) Klokov, *Betulaborysthenica* Klokov, *Stipacapillata* L., *Stipalessingiana* Trin. et Rupr., *Stipaucrainica* P. Smirn. and *Tulipabiebersteiniana* Schult. et Schult.f.s.l.) and 17 species in the ‘Red List of the Kherson Region’ (Kherson Regional Council 2013), i.e: *Amygdalusnana* L., *Bellevaliasarmatica* (Goergi) Woronow, *Centaureaadpressa* Ledeb., *Convallariamajalis* L., *Dianthusandrzejowskianus* (Zapal.) Kulcz., *Elytrigiapseudocaesia* (Pacz.) Prokudin, *Ephedradistachya* L., *Fraxinusexcelsior* L., *Irishalophyla* Pall., *Limoniumplatyphyllum* Linch., *Linariamacroura* (M.Bieb.) Chav., *Muscarineglectum* Guss., *Peucedanumruthenicum* M.Bieb., *Prangosodontalgica* (Pall.) Herrnat. et Heyn, *Quercusrobur* L., *Veronicacapsellicarpa* Dubovik and *Vincaherbacea* Waldst. et Kit. (Fig. 9).

The flora of the old cemeteries includes *Betulaborysthenica* Klokov, which was cultured and released, whereas *Paeoniatenuifolia* L., which is included in the Red Data Book of Ukraine (Didukh 2009) and *Anemonoidessylvestris* L., *Stachysgermanica* L. – included in the Red List of Kherson Region (Kherson Regional Council 2013) are plants that last only in the place of cultivation.

The sozophytes identified in the old cemeteries represented 19 families. The species represented the class *Magnoliophyta* (only one species belonged to the class *Pinophyta)*. The most frequent rare species in old cemeteries were: *Stipacapillata* L., and *Vincaherbacea* Waldst. et Kit. The shares of rare species in old cemeteries varied from 1.7% (Posad- Pokrovske) to 6% (Stanislav).

### Conclusions

The presence of typical steppe species and the large shares of native species compared to the aliens, indicate a relatively good state of preservation of steppe vegetation in old cemeteries in the Lower Dnipro region. The preservation of the natural vegetation cover in cemeteries is supported by the sacred status of cemeteries that are places where economic activities are not allowed. The old cemeteries can play an important role as places for protection of the steppe phytodiversity and provide the prospect of future activities for the local renewal of the steppe.

However, there are a number of adverse anthropogenic factors regarding old cemeteries that often reduce local steppe biodiversity. This is not only vandalism, but also the activities related with the care of graves (e.g. the removal of plant cover around graves or introduction of imported exotic, ornamental plants).

Taking the above into account, there is an urgent need for research on old cemeteries flora and its monitoring, as well as for creating nature reserves (in some of them), preserving their natural values.

## Figures and Tables

**Table 1.** General information about old cemeteries.

№	Name of the cemetery*^1^	Location	Year of establishment	Area (ha)	Landscape type*^3^	Way of using of the cemetery*^4^
1	Kherson Old Jewish cemetery	Kherson, the capital of the region	1870	2.58	U	Cl
2	Kherson Zabalka cemetery	Kherson, the capital of the region	18-19^th^ century	9.49	U	Cl
3	Kherson Memorial cemetery	Kherson, the capital of the region	1780	10.45	U	Cl
4	Ekonomiia Ivanivka	Kherson Region, Beryslav District	1855-1865	0.43	A	Ab
5	Kurgan near khutir Balakshova	Kherson Region, Beryslav District	1855-1865	0.7	A	Ab
6	Dolmativka	Kherson Region, Skadovsk District, v. Dolmativka	1850-1855	3.17	R	Cu
7	Nyzhni Torhai	Kherson Region, Henichesk District, v. Nyzhni Torhai	1840	1.1	R	Cu
8	Poniativka	Kherson Region, Kherson District, v. Poniativka	1780	1.1	R	Cu
9	Posad-Pokrovske	Kherson Region, Kherson District, v. Posad-Pokrovske	1789	3.6	R	Cu
10	Stanislav	Kherson Region, Kherson District, v. Stanislav	1697	6.79	R	Cu
11	Tokarivka	Kherson Region, Kherson District, v. Tokarivka	1780	2.81	A	Cu
12	Tryfonivka	Kherson Region, Beryslav District, v. Tryfonivka	1863	3.2	R	Cu
13	Tiahynka	Kherson Region, Beryslavsky District, v. Tiahynka	1778	5.86	R	Cu

**Table 2.** The basic parameters characterising the flora of old cemeteries in Lower Dnipro Region.

Characteristic of flora of investigated old cemeteries (OC)	The type of landscape in which the examined old cemeteries are located			Way of using of the cemetery area		
	Agricultural landscape	Rural landscape	Urban landscape	Abandoned OC	Closed OC	Current used OC
Number of investigated OC	3	7	3	2	3	8
Total number of species	229	360	228	154	360	369
Mean number of species per OC	126	166	190	107	166	165
Minimum and maximum number of species per OC	104-164	143-217	173-202	104-110	143-217	143-217
Number of OC with more than 150 species	1	5	3	0	5	5
Number of OC with more than 200 species	0	1	1	0	1	1
Number of native species	158 (69%)	226 (63%)	115 (50.4%)	112 (72.7%)	226 (63%)	235 (63.6%)
Number of steppe species	69 (30.1%)	96 (26.7%)	34 (15%)	42 (27.7%)	96 (26.7%)	103 (27.9%)
Number of rare species *in situ*	16 (7%)	16 (4.4%)	10 (4.4%)	8 (5.1%)	16 (4.4%)	18 (4.8%)
Number of rare species *ex situ*	2 (0.8%)	3 (0.8%)	1 (0.4%)	0 (0%)	3 (0.8%)	3 (0.8%)

**Table 3.** Additional characteristic of old cemeteries

№	Name of the cemetery*^1^	TNoS*^2^	NoNS*^2^	NoSS*^2^	NoRS (RDBU)*^2^	NoRS (KRRL)*^2^	NoAS*^2^	NoEg*^2^	DSS (km)*^2^
1	Kherson Old Jewish cemetery	195	102	29	1	4	93	19	5.38
2	Kherson Zabalka cemetery	173	93	24	2	2	80	16	6.7
3	Kherson Memorial cemetery	202	103	29	0	7	99	23	5.13
4	Ekonomiia Ivanivka	110	78	21	0	6	32	5	13.73
5	Kurgan near khutir Balakshova	104	80	28	1	1	24	3	13.55
6	Dolmativka	148	99	33	2	2	49	10	1.8
7	Nyzhni Torhai	144	90	33	2	2	54	11	2.84
8	Poniativka	143	84	25	2	4	59	14	1.2
9	Posad-Pokrovske	173	96	25	1	2	77	16	14.8
10	Stanislav	183	109	38	5	6	74	17	1.33
11	Tokarivka	164	114	48	2	7	50	12	0.2
12	Tryfonivka	155	101	33	6	2	54	12	10.7
13	Tiahynka	217	136	54	5	6	81	19	0.7

## Footnotes

*1 As cemeteries in villages did not have a name, we called them according to the nearest villages or historical place, in case absent the nearest village. Abbreviations: 1 – Kherson Old Jewish cemetery; 2 - Kherson Zabalka cemetery; 3 – Kherson Memorial cemetery; 4 - Ekonomiia Ivanivka; 5 - Kurgan near khutir Balakshova; 6 – Dolmativka; 7 - Nyzhni Torhai; 8 – Poniativka; 9 - Posad-Pokrovske; 10 – Stanislav; 11 – Tokarivka; 12 – Tryfonivka; 13 – Tiahynka.

*2 Abbreviations: TNoS - Total number of species; NoNS - Number of native species; NoSS - Number of steppe species; NoRS (RDBU) - Number of rare species (RDBU), NoRS (KRRL) - Number of rare species (KRRL); NoAS - Number of alien (or ruderal) species; NoEg - Number of ergasiophytes; DSS (km) - Distance to the nearest natural steppe site (km).

*3 Abbreviations of the old cemetery in landscape: A – agricultural landscape (blue colour in charts), R – rural landscape (green colour in charts), U – urban landscape (orange colour in charts).

*4 Abbreviations: the way of using cemetery area: Cu – currently used, Cl – closed, Ab – abandoned.
